# Hierarchical Reinforcement Learning for Quadrupedal Robots: Efficient Object Manipulation in Constrained Environments

**DOI:** 10.3390/s25051565

**Published:** 2025-03-04

**Authors:** David Azimi, Reza Hoseinnezhad

**Affiliations:** 1 School of Information Technology, Deakin University, Victoria 3125, Australia; dazimi@deakin.edu.au; 2 School of Engineering, RMIT University, Victoria 3082, Australia

**Keywords:** reinforcement learning, robotic manipulation, quadrupedal robots

## Abstract

This study introduces a hierarchical reinforcement learning (RL) framework tailored to object manipulation tasks by quadrupedal robots, emphasizing their real-world deployment. The proposed approach adopts a sensor-driven control structure capable of addressing challenges in dense and cluttered environments filled with walls and obstacles. A novel reward function is central to the method, incorporating sensor-based obstacle observations to optimize the decision-making. This design minimizes the computational demands while maintaining adaptability and robust functionality. Simulated trials conducted in NVIDIA Isaac Sim, utilizing ANYbotics quadrupedal robots, demonstrated a high manipulation accuracy, with a mean positioning error of 11 cm across object–target distances of up to 10 m. Furthermore, the RL framework effectively integrates path planning in complex environments, achieving energy-efficient and stable operations. These findings establish the framework as a promising approach for advanced robotics requiring versatility, efficiency, and practical deployability.

## 1. Introduction

Recent advancements in reinforcement learning (RL) have significantly contributed to the field of legged robot locomotion [1,2,3], as well as robotic hand manipulation [4,5]. Legged robots offer unique advantages due to their ability to traverse terrains inaccessible to wheeled robots. While many studies focus on obstacle avoidance, real-world applications often require robots to interact with and manipulate objects. This capability, referred to in the literature as locomanipulation, describes the integration of locomotion and object manipulation in mobile robots.

The current research on locomanipulation for legged robots primarily concentrates on quadrupedal robots equipped with robotic arms [6,7,8]. Other studies have explored the manipulation of spherical objects, focusing on achieving the desired velocity and direction [9,10,11]. Additionally, some works have investigated the use of a single leg for object manipulation [12,13,14].

Another emerging area of interest involves whole-body object manipulation, especially when dealing with objects comparable in size to the robot itself. For instance, Nachum et al. [15] proposed a hierarchical Sim2Real framework that separately trains low-level goal-reaching and high-level task-solving policies in simulation to move objects to target locations before transferring these skills to the real world. Similarly, Jeon et al. [16] introduced a hierarchical RL framework that enables quadrupedal robots to manipulate large objects using their entire bodies. This approach employs a high-level controller to generate velocity commands and a low-level controller to execute these commands through joint torque control.

In this paper, we present a novel hierarchical structure whose backbone is similar to that of the frameworks presented in [15,16]. However, unlike most existing studies that assume operation on open ground, we particularly devise our solution for applications where robots must function in cluttered environments with obstacles such as walls, which lead to the additional need for operation in confined spaces. We propose an enhanced reward function as part of the PPO algorithm that incorporates observations and accounts for the presence of walls, enabling the training of policies for robust operation in such constrained environments.

The remainder of this paper is structured as follows: Section 2 presents the problem statement and the theoretical foundations for reinforcement learning in object manipulation by quadrupedal robots. Section 3 describes the proposed hierarchical framework and reward function, including its implementation details. Section 4 discusses the experimental setup and the results obtained in simulated environments. Finally, Section 5 concludes with insights, the limitations of this study, and directions for future research.

## 2. Problem Statement

Consider a legged robot characterized by the following dynamic state vector:

(1) $$s^{\left(t\right)}={\left[P_{r}^{\left(t\right)}\;{\dot{P}}_{r}^{\left(t\right)}\;q_{r}^{\left(t\right)}\;{\dot{q}}_{r}^{\left(t\right)}\;j_{p}^{\left(t\right)}\;{\dot{j}}_{p}^{\left(t\right)}\right]}^{⊤},$$

which encompasses the robot’s global center position ($P_{r}$), velocity (${\dot{P}}_{r}$), orientation ($q_{r}$), and angular velocity (${\dot{q}}_{r}$), as well as the positions ($j_{p}$) and velocities (${\dot{j}}_{p}$) of its joints. Additionally, assume that the existence of an object, the position of its center, and *m* key points are known based on measurements, denoted as $P_{o}$. These measurements are acquired through external sensors, such as a calibrated camera employing object detection and coordinate transformations to the global frame. The object’s position can be estimated using a front-mounted RGB-D camera located on the robot’s torso, providing a direct line of sight to the object and obstacles ahead. The camera captures depth and RGB images, which are processed using an object detection algorithm based on convolutional neural networks (CNNs) to determine key object features. The positional data are then transformed into the global coordinate frame using the robot’s onboard odometry and inertial measurement unit (IMU) readings.

Note that $P_{o}$ is the measurement vector and not part of the state.

The task is to design a control mechanism that generates joint torque or positional commands $a^{\left(t\right)}\in R^{n}$ for *n* joints of the robot at time *t*. The subsequent system state evolves as per the relationship

(2) $$s^{\left(t+1\right)}=f\left(s^{\left(t\right)},a^{\left(t\right)},\eta^{\left(t\right)}\right),$$

where f(·) represents the robot’s dynamics transition function, and $\eta^{\left(t\right)}$ models noise or environmental disturbances. The robot’s pose adjustment is expected to impact the object’s pose through contact, which is referred to as body-assisted manipulation. The objective is to minimize the discrepancy between the object’s resulting pose $P_{o}$ and its target pose $P_{\mathrm{target}}$ while avoiding excessive action magnitudes to conserve energy. This problem can be expressed as the minimization of the following cost function:

(3) $$J=\sum_{t=0}^{T-1}∥P_{o}^{\left(t\right)}-P_{\mathrm{target}}{∥}^{2}+\lambda_{u}{∥a^{\left(t\right)}∥}^{2},$$

where $\lambda_{u}$ is a regularization parameter to penalize large actions. The objective function in Equation (3) follows a quadratic form similar to the standard Linear Quadratic Regulator (LQR) formulation, where control actions are penalized alongside the state deviation from the desired target. Classical LQR-based methods have been widely used in control systems for optimal regulation [17,18]. However, the LQR assumes known system dynamics and requires linearizable models, whereas reinforcement learning (RL) allows for direct policy learning in complex, high-dimensional environments where the system dynamics may be unknown or highly nonlinear.

To address the above control problem, we adopt a **model-free reinforcement learning** (RL) approach to determining the optimal policy $\pi (a^{\left(t\right)}|s^{\left(t\right)},P_{o}^{\left(t\right)},P_{w}^{\left(t\right)})$ that maximizes the cumulative reward R=−J, where the cost function *J* encapsulates the desired objectives of minimizing the position error and penalizing excessive actions. Note that $P_{o}^{\left(t\right)}$ and $P_{w}^{\left(t\right)}$ denote the measurements acquired from the sensors in relation to the location and orientation of the object and obstacles, respectively.

In this framework, the state space S is defined as

(4) $$S=\{s^{\left(t\right)},P_{o}^{\left(t\right)},P_{w}^{\left(t\right)}\},$$

where $s^{\left(t\right)}$ encapsulates the robot’s dynamics, including the joint positions and velocities, as well as the robot’s global position and orientation. The terms $P_{o}^{\left(t\right)}$ and $P_{w}^{\left(t\right)}$ provide the positional information on the object to be manipulated and the obstacles, respectively. These observations are obtained via external sensors. The action space A is defined as

(5) $$A=\{a^{\left(t\right)}\in R^{n}\},$$

where *n* is the number of joints in the robot, and $a^{\left(t\right)}$ represents the joint torque or position commands.

The reward function needs to be designed to reflect the objectives of the control problem. The policy $\pi (a^{\left(t\right)}|s^{\left(t\right)},P_{o},P_{w})$ is parameterized using a neural network with parameters θ. The policy outputs a probability distribution over actions $a^{\left(t\right)}$ given the current state and observations:

(6) $$\pi_{\theta }\left(a^{\left(t\right)}|s^{\left(t\right)},P_{o}^{\left(t\right)},P_{w}^{\left(t\right)}\right)=N\left(\mu_{\theta },\Sigma_{\theta }\right),$$

where $N(\mu_{\theta },\Sigma_{\theta })$ is a Gaussian distribution parameterized by the mean $\mu_{\theta }$ and covariance $\Sigma_{\theta }$ predicted by the neural network.

The choice of a Gaussian distribution for policy parameterization is common in continuous-action-space reinforcement learning. This distribution allows for smooth exploration by modeling stochasticity in the action selection, which is crucial for learning robust policies. The mean $\mu_{\theta }$ represents the most likely action, while the covariance $\Sigma_{\theta }$ governs exploration by introducing controlled variability into the actions. This stochastic policy formulation is particularly beneficial in robotics, as it enables adaptability in uncertain environments while preventing overly deterministic behaviors that could lead to poor generalization.

The objective of reinforcement learning is to optimize the policy $\pi (a^{\left(t\right)}|s^{\left(t\right)},P_{o}^{\left(t\right)},P_{w}^{\left(t\right)})$ such that the expected cumulative reward is maximized:

(7) $$L\left(\theta \right)=E_{\pi_{\theta }}\left[\sum_{t=0}^{T-1}r_{t}\right].$$

**Figure 1:**
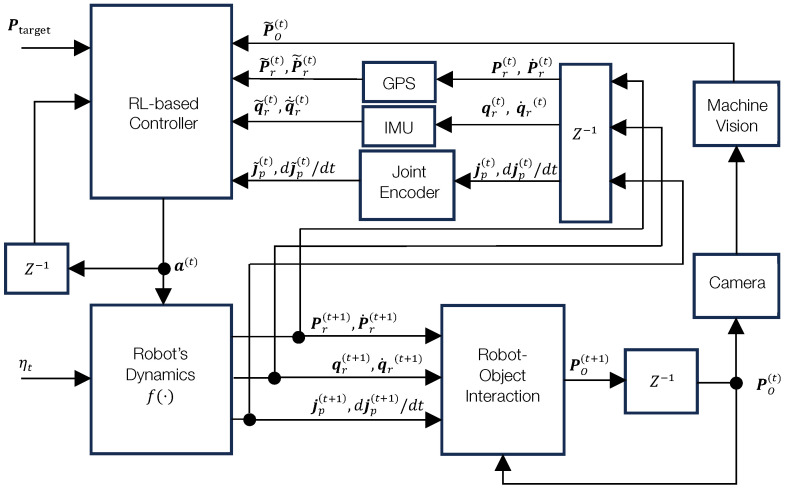
System block diagram illustrating the components of the robot control framework. The RL-based controller is the primary focus of this work.

The control framework for the legged robot, as depicted in Figure 1, consists of multiple interconnected subsystems responsible for perception, control, and actuation. At the core of this framework is the RL-based controller, which is designed to produce the optimal joint actions $a^{\left(t\right)}\in R^{n}$, leveraging sensory feedback and observations from the environment. Below, we detail the components and their roles in the control process:

1. Sensors: The control framework integrates multiple sensory inputs, including an RGB-D camera for object and obstacle detection, an IMU for orientation estimation, and joint encoders for proprioception. These provide the necessary state observations for reinforcement-learning-based decision-making.
2. RL-based controller: The controller computes the optimal actions $a^{\left(t\right)}$ conditioned on the current state of the robot, as represented in Equation (1). Additionally, the controller utilizes object and obstacle measurements $P_{o}^{\left(t\right)}$ and $P_{w}^{\left(t\right)}$ to make decisions.
3. Robot dynamics: The state evolves according to the dynamics described in Equation (2), where f(·) represents the robot’s dynamics transition function.
4. Robot–object interaction: The change in the robot’s pose, resulting from the applied actions, directly influences the object’s position $P_{o}^{\left(t+1\right)}$, establishing coupling between the robot’s actions and object manipulation.

## 3. The Proposed Solution

**Figure 2:**
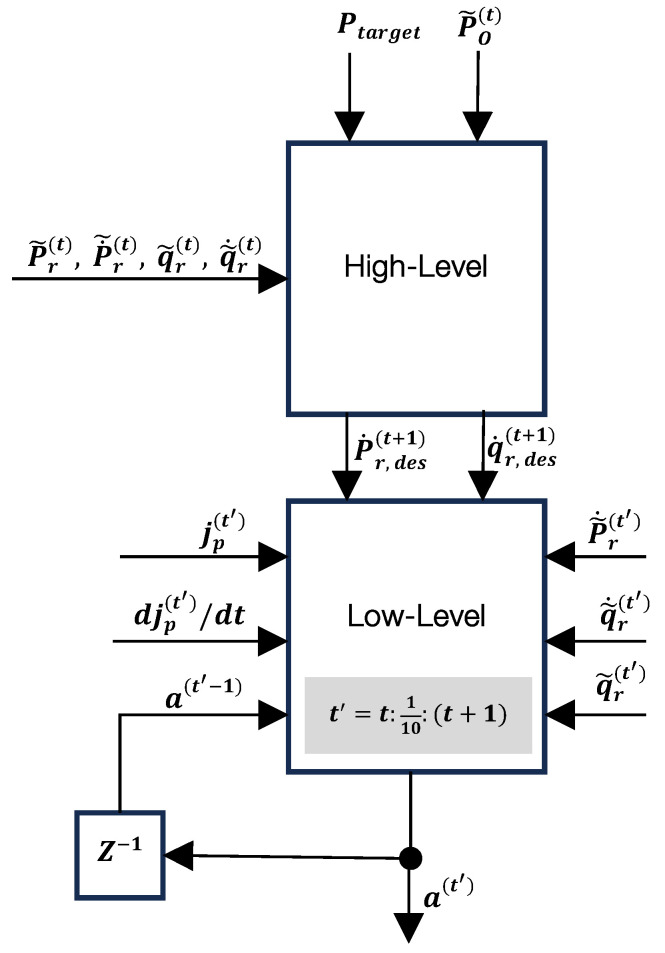
The proposed two-level controller design for the contents of the RL-based controller block in Figure 1.

Our preliminary experiments, along with findings from other studies [16], demonstrate that a single policy is insufficient to effectively navigate an object to its target pose. Given the complexity of the manipulation task, we employ a hierarchical RL framework (see Figure 2). The control framework employs a hierarchical RL approach, where the high-level policy determines the robot’s target motion based on the task objectives, and the low-level policy translates these into joint commands for execution. This layered strategy improves the robustness and stability in complex environments.

A key distinction between the two controllers lies in their operational frequencies. The low-level controller operates at a much higher frequency than that of the high-level controller. Specifically, the high-level controller generates outputs every 200 ms (corresponding to a frequency of 5 Hz), while the low-level controller achieves these desired velocities through a sequence of 10 joint action vectors (with each consisting of 12 dimensions for a quadrupedal robot) executed at a frequency of 50 Hz. Figure 2 provides a detailed view of the RL-based controller block from Figure 1. The distinction in the sampling rates is depicted through the use of the time notations *t* and $t^{\prime }$.

To be precise, there are two clocks in place: one indexed at times denoted by *t* that synchronizes the processes at the high-level controller and the other that starts at each time *t*, indexed at times denoted by $t^{\prime }=t:\frac{1}{n}:\left(t+1\right)$, that synchronizes the processes at the low-level controller. In our experiments, n=10. At each time $t^{\prime }$, the *low-level* controller generates the final control actions $a^{\left(t^{\prime }\right)}$ which are applied to the joint actuators. The inputs to this controller include the joint and body positions and velocities obtained from the sensors at times $t^{\prime }$ (illustrated in Figure 1), along with the next desired body velocity commands ${\dot{P}}_{r,\mathrm{des}}^{\left(t+1\right)}$ and ${\dot{q}}_{r,\mathrm{des}}^{\left(t+1\right)}$ provided by the high-level controller.

**Figure 3:**
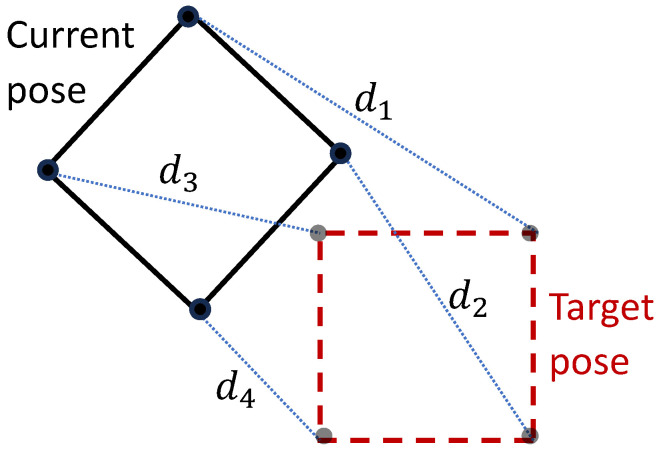
The distance between the object’s current pose and its target pose is calculated based on the four distances between keypoints on the object and their corresponding points at the target location.

The low-level controller is tasked with converting these desired velocity commands into specific joint actions. To achieve this, we propose using a deep fully connected neural network for the transformation process. The high-level policy is trained to optimize

(8) $$r_{t}^{\mathrm{high}}=-{∥P_{o}^{\left(t\right)}-P_{\mathrm{target}}∥}^{2},$$

where the distance is computed in terms of the distances from keypoints on the object to their corresponding points in the target location (see Figure 3). The low-level policy optimizes

(9) $$r_{t^{\prime }}^{\mathrm{low}}=-∥{\dot{P}}_{r,\mathrm{des}}^{\left(t+1\right)}-{\tilde{\dot{P}}}_{r}^{\left(t^{\prime }\right)}{∥}^{2}-{∥{\dot{q}}_{r,\mathrm{des}}^{\left(t+1\right)}-{\tilde{\dot{q}}}_{r}^{\left(t^{\prime }\right)}∥}^{2}.$$

**Figure 4:**
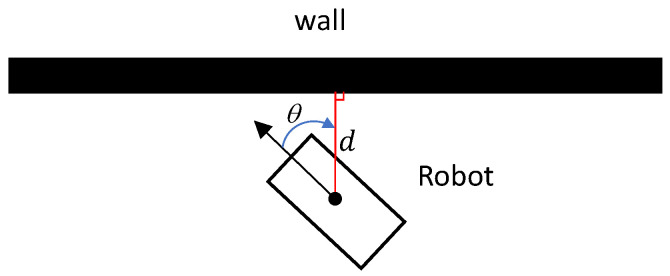
The distance and orientation angle of the robot with respect to the closest wall are measured using the vision sensor and used to augment the reward function in the high-level controller.

While most prior research assumes operation in open environments, our work is centered on enabling robots to operate in confined spaces with obstacles such as walls and corners. To our knowledge, this is the first study to directly address object manipulation in such constrained settings. To achieve this, we incorporate measurements of the robot’s distance and heading angle relative to the nearest wall or obstacle, as depicted in Figure 4. Specifically, we introduce an additional component into the high-level policy reward that penalizes the robot heavily when it approaches closer than a defined threshold to a wall and its heading angle falls below a certain value. This mechanism discourages movements in potentially hazardous directions, thus enhancing safety.

In scenarios where occlusions occur—such as when an object is partially hidden behind another obstacle—the system compensates using a Kalman filter to estimate the object’s position based on previous frames. If an object is fully occluded for an extended period, the robot performs exploratory movements to improve its visibility. Additionally, the measurements from the IMU and proprioceptive sensors help refine object localization by detecting contact events, providing additional cues for position estimation.

The high-level controller processes data from various sensors, including the object positions detected by vision sensors, to determine the optimal next steps for the robot. The outputs of the high-level controller are the desired linear velocity of the robot’s center of mass, ${\dot{P}}_{r,\mathrm{des}}$, and the desired angular velocity, ${\dot{q}}_{r,\mathrm{des}}$. Similar to the low-level controller, the high-level controller employs a deep fully connected neural network to map the sensory data to the desired robot motions.

Both the high-level and low-level controllers are implemented as fully connected deep networks, trained using the Proximal Policy Optimization (PPO) algorithm. Originally introduced in 2017 by Schulman et al. [19], PPO has become a widely used method for training legged robots due to its computational efficiency and robustness [2,20,21]. PPO works by maximizing a surrogate objective function given as follows:

(10) $$L^{\mathrm{PPO}}\left(\theta \right)=E_{\pi_{\theta }}\left[\min\left(r_{t}\left(\theta \right){\hat{A}}_{t},\mathrm{clip}\left(r_{t}\left(\theta \right),1-\epsilon ,1+\epsilon \right){\hat{A}}_{t}\right)\right],$$

where $r_{t}\left(\theta \right)$ represents the ratio of the probabilities between the updated and previous policies, ${\hat{A}}_{t}$ is the computed advantage, and ϵ is a parameter introduced to restrict excessive policy updates.

To provide a clearer understanding of the reinforcement learning process used in our framework, we include a pseudo-code representation of the PPO algorithm in Algorithm 1. This pseudo-code outlines the iterative procedure of data collection, advantage estimation, policy optimization, and value function updates. The clipping mechanism in the PPO objective prevents excessively large policy updates, ensuring stable learning and efficient convergence.
**Algorithm 1** Proximal Policy Optimization (PPO)**Require:** Initialize the policy parameters θ and the value function parameters ϕ  **while** not converged, **do**   **for** each training iteration, **do**    **for** each environment step, **do**      $s_{t}\leftarrow$ current state      $a_{t}\leftarrow$ action sampled from policy $\pi_{\theta }$      $r_{t}\leftarrow$ reward received      $s_{t+1}\leftarrow$ next state after executing $a_{t}$      Store $\left(s_{t},a_{t},r_{t},s_{t+1}\right)$ in buffer    **end for**    compute the advantage estimates ${\hat{A}}_{t}$ using Generalized Advantage Estimation (GAE)    **for** each policy update, step **do**      $L\left(\theta \right)\leftarrow E\left[\min\left(r_{t}\left(\theta \right){\hat{A}}_{t},\mathrm{clip}\left(r_{t}\left(\theta \right),1-\epsilon ,1+\epsilon \right){\hat{A}}_{t}\right)\right]$      update policy θ using gradient ascent on L(θ)$L_{V}\left(\phi \right)\leftarrow {\left(V_{\phi }\left(s_{t}\right)-R_{t}\right)}^{2}$      update value function ϕ by minimizing $L_{V}\left(\phi \right)$    **end for**   **end for**  **end while**

To train the low-level controller, we use randomly generated desired linear and angular velocity inputs for the robot’s center of mass. The PPO algorithm employs the following cost function:

(11) $$J_{\mathrm{low}}=\sum_{t^{\prime }=0}^{T-1}\left(\frac{\left|\right|{\dot{P}}_{r,\mathrm{des}}^{\left(t^{\prime }\right)}-{\tilde{\dot{P}}}_{r}^{\left(t^{\prime }\right)}{\left|\right|}^{2}}{v_{\max}^{2}}+\frac{\left|\right|{\dot{q}}_{r,\mathrm{des}}^{\left(t^{\prime }\right)}-{\tilde{\dot{q}}}_{r}^{\left(t^{\prime }\right)}{\left|\right|}^{2}}{\omega_{\max}^{2}}\right)$$

Here, $v_{\max}$ and $\omega_{\max}$ are the respective maximum linear and angular velocities used to normalize the terms. The parameter *T* denotes the total number of training iterations. To maintain consistency in training, random desired velocity inputs are generated and held constant for every 10 iterations, resulting in N=⌈T/10⌉ distinct sets of desired inputs. For each time step $t^{\prime }$, the input velocities correspond to the set indexed by t=⌈T/10⌉−1.

The robot’s velocity and angular speed values, ${\tilde{\dot{P}}}_{r}^{\left(t^{\prime }\right)}$ and ${\tilde{\dot{q}}}_{r}^{\left(t^{\prime }\right)}$, used in Equation (11), are indirectly related to the action commands $a^{\left(t^{\prime }\right)}$ generated by the low-level controller. These values depend on the robot’s inherent dynamics, which are not explicitly known. Reinforcement learning is employed here to enable the controller to achieve an accurate and stable performance by implicitly learning the robot’s dynamics over repeated training iterations using the PPO algorithm.

For the high-level controller, the following cost function is used in the PPO algorithm:

(12) $$J_{\mathrm{high}}=\sum_{t=0}^{N-1}\left(\frac{\left|\right|{\tilde{P}}_{o}^{\left(t\right)}-P_{\mathrm{target}}{\left|\right|}^{2}}{d_{\max}^{2}}+\lambda \left[\frac{\left|\right|{\tilde{\dot{P}}}_{r}^{\left(t\right)}{\left|\right|}^{2}}{v_{\max}^{2}}+\frac{\left|\right|{\tilde{\dot{q}}}_{r}^{\left(t\right)}{\left|\right|}^{2}}{\omega_{\max}^{2}}\right]\right)$$

In this formulation, λ is a regularization parameter that discourages large velocity commands, promoting smoother motion and energy efficiency. The terms $v_{\max}$ and $\omega_{\max}$ represent the maximum allowable velocities, as defined earlier, while $d_{\max}$ specifies the maximum distance between the robot and the target, a parameter tailored to the application requirements.

The cost function in Equation (3) represents a global objective that penalizes deviations in the object positioning while minimizing excessive control actions. However, direct optimization of this function in a single policy can be inefficient, particularly in complex environments. To facilitate structured learning, we decompose *J* into two components:

- $J_{\mathrm{high}}$, given in Equation (12), which governs high-level motion planning, focusing on guiding the robot towards effective object manipulation while ensuring obstacle avoidance and task efficiency;
- $J_{\mathrm{low}}$, given in Equation (11), which regulates low-level control by ensuring accurate tracking of the high-level commands, enforcing smooth and stable actuation.

This hierarchical decomposition allows each policy to specialize in a distinct aspect of control, reducing the complexity of direct policy optimization while improving the stability and sample efficiency. This approach aligns with hierarchical reinforcement learning principles, where higher layers handle strategic decisions while lower layers execute fine-grained actions.

The weight values for different reward components were determined through iterative empirical tuning, balancing the object positioning accuracy, obstacle avoidance, and motion smoothness. These values were adjusted based on prior research on hierarchical RL for legged robots [16] and optimized to ensure convergence and stability in training.

## 4. Task Description and Simulation Results

**Figure 5:**
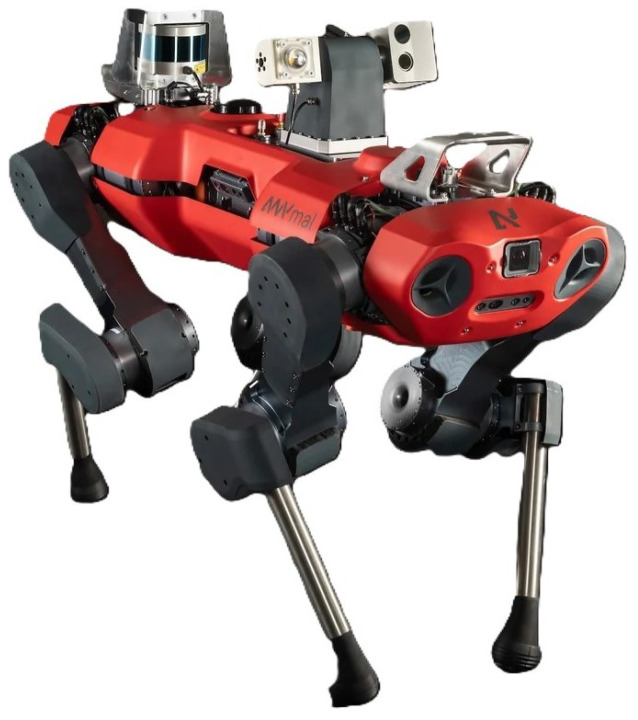
The “Anymal C” robot by ANYbotics, simulated in our experiments.

For our experimental setup, we employed the Isaac Lab framework, an advanced tool created by NVIDIA to support reinforcement learning for robotics [22]. This framework is built on NVIDIA’s Isaac Sim, a high-fidelity simulation platform tailored to the development, simulation, and testing of robotic systems [23]. Within this framework, users can choose from various quadrupedal robot models that differ in their size, mass, and locomotion capabilities. For our experiments, we selected the Anymal C quadrupedal robot (depicted in Figure 5) due to its compact yet highly stable design, making it versatile for a wide range of tasks. The robot has a weight of approximately 50 kg and a height of about 0.6 m in its standard configuration. Its body dimensions, roughly 1.05 m × 0.52 m, strike an excellent balance between agility and stability, ensuring suitability for diverse robotic applications.

The object utilized in the experiments was a cube with dimensions of 60cm×60cm×60cm. At the beginning of each episode, the cube was positioned at a random distance from the robot, uniformly sampled within the range of 1–2 m. Similarly, the target position was randomly chosen within a 10 m radius from the robot. Both the initial orientations of the robot and the object were randomized. Additionally, to further enhance the robustness, different surface frictions and cluttered environments were included in the simulation setup, ensuring the learned policies generalize well to a variety of real-world conditions. This setup introduced diverse conditions during training, enabling the control system, implemented as fully connected neural networks, to learn to adapt effectively through reinforcement learning.

The neural networks used for the high-level and low-level controller blocks (depicted in Figure 2) were fully connected, with the high-level network comprising two hidden layers and the low-level network having three hidden layers, with each containing 128 neurons. Exponential Linear Unit (ELU) activation functions were employed to connect the layers, defined as follows:

(13) $$f\left(x\right)=\left\{\begin{array}{cc} x & \mathrm{if}\;x>0,\\ \alpha (e^{x}-1) & \mathrm{if}\;x\leq 0, \end{array}\right.$$

where the parameter α was fixed at a value of 1.5.

As outlined in Section 3, the Proximal Policy Optimization (PPO) algorithm was utilized to train the neural networks within the reinforcement learning framework. Several repositories provide baseline implementations of PPO and related algorithms, optimized for robotic control tasks. Among these, NVIDIA’s rl-pytorch, part of the Isaac SDK ecosystem, offers highly efficient tools for implementing robotic reinforcement learning [24].

Another advanced framework, rsl_rl, extends rl-pytorch with enhanced capabilities for diverse applications in robotic control and simulation [25]. This framework offers several benefits, including support for highly parallelized training and seamless integration with GPU-accelerated environments, making it especially effective for training deep learning models in complex control scenarios. For our experiments, we utilized rsl_rl with the parameter settings specified in Table 1.

In the low-level policy, we used the desired linear and angular velocities, combined with proprioceptive data, as the input observations. The policy’s outputs are the joint target positions, which are subsequently processed by a Proportional–Derivative (PD) controller to calculate the required joint torques. This policy architecture is inspired by the approach outlined in [1]. The observations for the low-level policy consist of the robot’s linear and angular base velocities, the gravity vector, and the joint positions and velocities, as well as the action taken in the previous time step. The reward function primarily aims to ensure accurate tracking of the commanded linear and angular velocities, while additional penalties are applied to undesired vertical (z-axis) velocities, as well as roll and pitch angular velocities. Furthermore, the rewards discourage excessive changes in actions, joint velocities, and accelerations, promoting smoother motion, lowering the power usage, and minimizing the wear on the hardware caused by sudden changes in dynamics.

Training of the low-level policy was conducted in a simulation using 4096 parallel robot instances. Policy updates were applied every 24 steps, and training was terminated after 1500 iterations. The policy was modeled using a fully connected neural network with three hidden layers comprising 512, 256, and 128 nodes, respectively.

Once the low-level policy was trained with randomized velocity commands, it was frozen, and training of the high-level policy commenced. The high-level policy, also implemented as a fully connected neural network, features two hidden layers, with each containing 128 nodes. The inputs for the high-level policy include the commanded base velocity, the robot’s base pose and velocities, the gravity vector, the relative distance and angle to nearby walls, the current object pose, and the target object pose.

Selecting appropriate weights for the rewards based on the errors in the center-of-mass position and orientation has proven to be a significant challenge, often yielding suboptimal outcomes. To address this, we adopted the key-point-based reward strategy described in [16]. This approach minimizes the Euclidean distance between the actual and target key points of the object, eliminating the need for explicit orientation error terms. The main reward for the high-level policy encourages reducing the distance between the key points of the object and its target pose. Additionally, an intrinsic reward motivates the robot to approach the object by rewarding proximity between the robot’s center of mass and the object’s center of mass. To enhance the policy’s stability, penalties are imposed for abrupt changes in actions and joint velocities, reducing the power consumption and mitigating hardware strain.

The use of domain randomization, including variations in the sensor noise, object properties, and environmental layouts, further strengthens the adaptability of the learned policies, mitigating the need to explicitly add more training scenarios. Training was conducted over a maximum of 12,500 iterations, with each environment running for 8 steps per iteration. The model weights were saved at intervals of 50 iterations for analysis and backup purposes.

**Figure 6:**
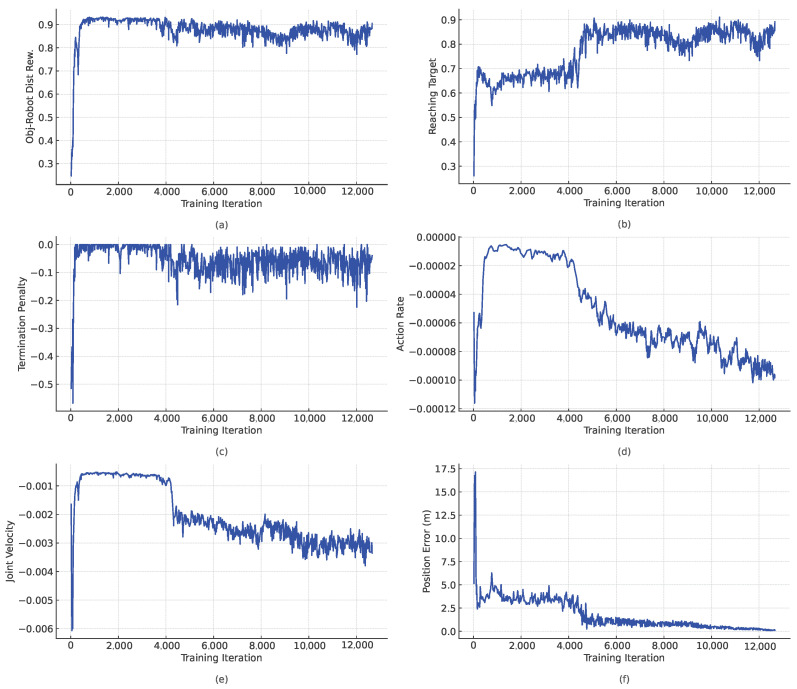
Convergence of various indicators during training: (**a**–**e**) Training rewards associated with the object–robot distance, the object–target distance, episode termination (to avoid falls), action rate (for smooth transitions between actions), and joint velocities to ensure minimal power consumption, respectively. (**f**) The controller’s performance in terms of the final distance between the object and target positions.

To further illustrate the framework’s performance, Figure 6 presents key training metrics, including the position error, termination penalties, and velocity adjustments. These plots provide insight into the robot’s learning process and convergence behavior. Additionally, a Supplementary Video demonstrating the robot’s training and execution in simulation was included to help visualize the object manipulation process in constrained environments. The outcomes of the training process are depicted in Figure 6. The results reveal convergence towards an optimal set of network parameters, enabling object positioning with a final positioning error of approximately 11 cm—a notable result given the initial distances of around 10 m.

Throughout the training process, the position error consistently decreased, demonstrating the agent’s progressive refinement in aligning the object’s position with the desired target. Larger errors are observed during the initial phases of training but diminish as the agent acquires more effective control strategies.

The reward for reaching the object exhibited a steady increase, signifying the agent’s enhanced capability to navigate toward the object over time. Similarly, the rewards related to guiding the object to the target position improved progressively, reflecting the agent’s growing precision in completing the manipulation task.

To assess the sensitivity of the reward function, we conducted initial trials with varied weightings. We observed that increasing the weight for obstacle avoidance significantly improved safe navigation but at the cost of slightly slower task completion. Conversely, reducing this weight led to more direct paths but higher collision rates. The final weight values were selected to achieve a balance between safety and efficiency.

Over the course of training, the penalties related to the termination, joint velocity, and action rate steadily decreased (corresponding to an increase in their respective rewards), indicating improved stability and energy efficiency. The decline in the termination penalties highlights the agent’s ability to avoid actions that might lead to premature episode termination. Moreover, the reduction in the penalties associated with the joint velocity and action rate demonstrated that the agent’s actions became smoother and more controlled as the training advanced.

The proposed framework demonstrates strong performance in terms of its positioning accuracy and energy efficiency, achieving a final object positioning error of 11 cm over target distances of up to 10 m. While direct numerical comparisons with the state-of-the-art methods are not presented due to differences in the problem settings and training environments, we note that our approach aligns with the performance levels reported in recent hierarchical RL-based locomotion and manipulation studies [1,16]. For example, Jeon et al. [16] achieved comparable task success rates in quadrupedal whole-body manipulation, while other works in deep RL-based locomotion [1] have reported a similar trajectory tracking accuracy. The primary distinction of our method lies in its focus on object manipulation in constrained spaces, a scenario not extensively addressed in prior work. Future research will aim to conduct direct comparisons with the baseline algorithms under identical experimental conditions.

## 5. Conclusions

This study has introduced an innovative hierarchical reinforcement learning framework specifically designed for quadrupedal robots tasked with object manipulation in challenging, obstacle-filled environments. By leveraging a sensor-driven control mechanism, this framework addresses the complexities of navigation and manipulation without relying on conventional grippers or simulation-restricted data.

The key contributions of this work include the development of an enhanced reward function tailored to dense and cluttered spaces, as well as the integration of efficient, fully connected deep learning models for both high-level decision-making and low-level control. These advancements not only reduce the computational demands but also ensure robustness and adaptability in real-world applications.

Experimental evaluations using simulated environments demonstrated the efficacy of the proposed framework, achieving high levels of precision in object positioning and energy efficiency. The results highlight the potential of this approach to enhance the locomanipulation capabilities in scenarios requiring simultaneous mobility and interaction with objects.

### Limitations and Future Directions

While the proposed framework demonstrates effective object manipulation in cluttered environments, several challenges remain. First, the current approach assumes static obstacles, whereas real-world environments often include dynamic elements. Future research could incorporate predictive models or real-time re-planning strategies to enhance the adaptability to moving obstacles.

Second, our work focuses on manipulating a single object at a time. Extending the framework to multi-object manipulation, particularly in scenarios requiring coordinated interactions, is an important direction for further development. Recent advancements in hierarchical reinforcement learning and multi-agent systems offer promising methodologies for addressing such challenges.

Additionally, enhancing real-world deployment by integrating domain adaptation techniques would improve the generalization beyond the simulated environment. Methods such as adversarial domain adaptation, as explored in [26], could be incorporated to bridge the sim-to-real gap more effectively.

Future work will aim to extend this framework to more complex and dynamic environments, incorporate additional sensory modalities, and validate its performance using physical hardware in real-world deployments. This research represents a significant step toward more capable, versatile, and practical robotic systems for diverse applications.

## Figures and Tables

**Table 1:** PPO algorithm parameters used in the experiment.

Parameter	Value
Value loss coefficient (value_loss_coef)	1.0
Clipped value loss (use_clipped_value_loss)	True
Clipping parameter (clip_param)	0.2
Entropy coefficient (entropy_coef)	0.005
Number of learning epochs (num_learning_epochs)	5
Number of mini-batches (num_mini_batches)	4
Learning rate (learning_rate)	$1.0\times 10^{-3}$
Discount factor (γ)	0.99
GAE lambda (λ)	0.95
Desired KL divergence (desired_kl)	0.01
Maximum gradient norm (max_grad_norm)	1.0

## Data Availability

No new data were created.

## Supplementary Materials

The following supporting information can be downloaded at https://www.mdpi.com/article/10.3390/s25051565/s1. Video S1: A sample of the learning process for the robot, visualized through simulations.
